# A pneumococcal controlled human infection model in Malawi: Transfer of an established pneumococcal carriage model from Liverpool, UK to Blantyre, Malawi – A feasibility study

**DOI:** 10.12688/wellcomeopenres.15689.2

**Published:** 2020-04-14

**Authors:** Ben Morton, Sarah Burr, Kondwani Jambo, Jamie Rylance, Marc Y.R. Henrion, Ndaziona Peter Banda, Edna Nsomba, Blessings Kapumba, Lucinda Manda-Taylor, Clemens Masesa, Daniela Ferrreira, Stephen B. Gordon, MARVELS Consortium

**Affiliations:** 1 Lung Health Group, Malawi-Liverpool Wellcome Trust Clinical Research Programme, Blantyre, PO Box 30096, Malawi; 2 Department of Clinical Sciences, Liverpool School of Tropical Medicine, Liverpool, L3 5QA, UK; 3 Critical Care Medicine, Liverpool University Hospitals NHS Foundation Trust, Liverpool, L9 7AL, UK; 4 Department of Medicine, Queen Elizabeth Central Hospital, Blantyre, PO Box 95, UK; 5 University of Malawi College of Medicine, Blantyre, Malawi

**Keywords:** Streptococcus pneumoniae, Pneumococcal carriage, Experimental medicine, pneumonia, vaccine, controlled human infection model, nasal colonisation, global health

## Abstract

*Streptococcus pneumoniae* is the leading cause of morbidity and mortality due to community acquired pneumonia, bacterial meningitis and bacteraemia worldwide. Pneumococcal conjugate vaccines protect against invasive disease, but are expensive to manufacture, limited in serotype coverage, associated with serotype replacement and demonstrate reduced effectiveness against mucosal colonisation.  As asymptomatic colonisation of the human nasopharynx is a prerequisite for pneumococcal disease, this is proposed as a marker for novel vaccine efficacy. Our team established a safe and reproducible pneumococcal controlled human infection model at Liverpool School of Tropical Medicine (LSTM). This has been used to test vaccine induced protection against nasopharyngeal carriage for ten years in over 1000 participants.

We will transfer established standardised operating procedures from LSTM to Malawi and test in up to 36 healthy participants. Primary endpoint: detection of the inoculated pneumococci by classical culture from nasal wash recovered from the participants after pneumococcal challenge. Secondary endpoints: confirmation of robust clinical and laboratory methods for sample capture and processing. Tertiary endpoints: participant acceptability of study and methods. We will test three doses of pneumococcal inoculation (20,000, 80,000 and 160,000 colony forming units [CFUs] per naris) using a parsimonious study design intended to reduce unnecessary exposure to participants. We hypothesise that 80,000 CFUs will induce nasal colonisation in approximately half of participants per established LSTM practice.

The aims of the feasibility study are: 1) Establish *Streptococcus pneumoniae* experimental human pneumococcal carriage in Malawi; 2) Confirm optimal nasopharyngeal pneumococcal challenge dose; 3) Confirm safety and measure potential symptoms; 4) Confirm sampling protocols and laboratory assays; 5) Assess feasibility and acceptability of consent and study procedures. Confirmation of pneumococcal controlled human infection model feasibility in Malawi will enable us to target pneumococcal vaccine candidates for an at-risk population who stand the most to gain from new and improved vaccine strategies.

## Abbreviations

AE: Adverse Event; CAP: Community Acquired Pneumonia; CFU: Colony Forming Units; CHIM: Controlled Human Infection Model; CRF: Case Report Form; DSMB: Data, Safety and Monitoring Board; EHPC: Experimental Human Pneumococcal Colonisation; ELISA: Enzyme-Linked Immunosorbent Assay; ELISPOT: Enzyme-Linked Immune Absorbent Spot; FBC: Full Blood Count; GCP: Good Clinical Practice; HIV: Human Immunodeficiency Virus; HTS: Human immunodeficiency virus Testing Service; ICH-GCP: International Conference on Harmonisation of Good Clinical Practice; LSTM: Liverpool School of Tropical Medicine; MARVELS: Malawi Accelerated Research in Vaccines, Experimental and Laboratory Systems; MHRA; Medicines and Healthcare products Regulatory Authority (UK Regulator); MK: Malawian Kwacha; MLW: Malawi-Liverpool Wellcome Trust Clinical Research Programme Laboratories; MTA: Material Transfer Agreement; NHSRC: National Health Sciences Research Committee; OM: Otitis Media; PBMC: Peripheral Blood Mononuclear Cell; PCR: Polymerase Chain Reaction; PCV: Pneumococcal conjugate vaccines; PCV13: Pneumococcal conjugate vaccine 13; PCVPA: Pneumococcal Carriage in Vulnerable Populations in Africa; PMPB: Pharmacy, Medicines and Poisons Board; QECH: Queen Elizabeth Central Hospital; RNA: Ribonucleic Acid; SAE: Serious Adverse Event; SOP: Standardised Operating Procedure; SUSAR: Serious Unexpected Serious Adverse Reaction; TSC: Trial Steering Committee.

## Introduction

*Streptococcus pneumoniae* is the leading cause of morbidity and mortality due to community acquired pneumonia (CAP), bacterial meningitis and bacteraemia worldwide ^1^. Pneumococcal infections cause over one million pneumonia deaths per year in children in the developing world and are also a major burden of otitis media globally. Pneumococcal conjugate vaccines (PCV) represent a great achievement in providing protection from invasive pneumococcal disease, but they are expensive to manufacture, limited in serotype coverage, associated with serotype replacement and are less effective against mucosal infection (13–20%) than against invasive disease ^2^. Alternative vaccine strategies are therefore still urgently needed ^3^ and several promising vaccine alternatives are being developed worldwide.

A major roadblock to the process of developing new protein vaccines has been a means of prioritising between proposed vaccine candidates ^4^. Because asymptomatic colonisation of the human nasopharynx is a prerequisite for pneumococcal disease, it has been proposed as a marker for vaccine efficacy ^5^. We established a safe and reproducible Controlled Human Infection Model (CHIM) at Liverpool School of Tropical Medicine (LSTM), U.K., which has been used to test the protection induced by vaccination against nasopharyngeal carriage ^6^. The Liverpool CHIM, also known as Experimental Human Pneumococcal Carriage (EHPC), established over the last ten years, uses a serotype 6B pneumococcal strain to establish carriage in 50% of healthy participants after nasopharyngeal challenge with 80,000 bacterial colony forming units presented to each nostril (naris) in 100 µl of saline. This model has been used to test the effect of new candidate vaccines with significant cost and time savings compared with phase III trials ^6^.

Despite the introduction of the PCV13 vaccine to Malawi in 2011, pneumococcal disease remains the single most important bacterial infection of adults and children ^7^. Long term follow-up has been rigorously carried out by the Pneumococcal Carriage in Vulnerable Populations in Africa (PCVPA) consortium ^8^. These data demonstrate that despite an impressive reduction in invasive pneumococcal disease for vaccinated children, there is persistent nasal carriage of both vaccine type and non-vaccine type *Streptococcus pneumoniae* in both children and HIV-infected adults ( Table 1). The implications of this important finding are that (A) herd effects with PCV13 are not as strong as in Malawi compared to US data, with vaccine type pneumococcus a continued threat to vulnerable adults and children and (B) the persistent pneumococcal carriage may indicate sub-optimal or short duration of PCV13 vaccine-induced immunity. There is therefore an urgent need for new vaccines in Malawi and EHPC may offer a means to choose among alternative novel vaccines.

**Table 1.** Pneumococcal Carriage in Vulnerable Populations in Africa (PCVPA) consortium data from 2018 on pneumococcal carriage and pneumococcal conjugate vaccine 13 status ^8^. The data show that despite vaccination, children aged 3–6 years have the same vaccine type carriage rates as unvaccinated 5–10-year olds.

Malawian PCVPA surveillance data	Children 3–6 years (PCV vaccinated)	Children 5–10 years (not PCV vaccinated)	HIV infected adults (not PCV vaccinated)
Vaccine type carriage	20.1% (95% CI: 18.0, 22.3)	20.1% (95% CI: 17.5, 22.9)	13.9% (95% CI:11.8, 16.2)
Non-vaccine type carriage	55.9% (95% CI: 53.3, 58.6)	36.8% (95% CI: 33.6, 40.0)	29.5% (95% CI: 26.7, 32.5)

A valid criticism of current vaccine research strategies is that these have been conducted in geographical areas and participant populations not at significant risk of disease. To address this, we propose to transfer our safe and established standardised operating procedures from the Liverpool CHIM to the Malawian setting. The Liverpool CHIM has been used in over 1000 healthy adults for the past nine years safely with no cases of active infection or related serious adverse effects. A Malawian pneumococcal CHIM will allow targeted pneumococcal vaccine candidate choices to be made based on relevant pathogen challenge in an at-risk population who stand the most to gain from new and improved vaccine strategies.

### Ethical considerations for controlled human infection studies in low income settings

The ethical considerations for CHIM studies and for experimental human pneumococcal carriage in particular were extensively explored at a workshop in Malawi from which a guideline summary was published ^9^. Further consideration of this topic was presented at an ASTM&H symposium in New Orleans, 2018. Briefly, the benefit of vaccine development in the target population has been overwhelmingly demonstrated by the malaria programme and further benefit is expected for vaccines deployed in populations with high exposure. The ethical principles governing CHIM studies are no different in low income countries than anywhere else but the operational challenges to ensure participant safety may involve more health services support than in highly resourced settings. Appropriate consent requires a high level of participant involvement and comprehension in any context - this may also require additional effort in settings where participants have experienced limited science education. The detailed consideration of community knowledge and views regarding the CHIM of pneumococcal carriage in Malawi will shortly be published (IN PRESS: BMC Medical Ethics ^10^).

## Study protocol

### Study aims

The aims of this study are:

1. Establish controlled human infection with *Streptococcus pneumoniae* serotype 6B in Malawi
2. Confirm nasopharyngeal pneumococcal challenge dose required to establish ~50% carriage in Malawian participants
3. Confirm safety and measure potential symptoms of controlled human infection procedures for study participants
4. Confirm sampling protocols and laboratory assays for immunological assessment relevant to vaccine testing.
5. Assess the feasibility and acceptability of consent and study procedures for participants.

### Study hypothesis

That inoculation of Malawian volunteers with 80,000 cfu/naris will safely result in nasopharyngeal carriage in ~50% of participants as it has done in Liverpool.

### Study design

A feasibility study of adult healthy human participants experimentally exposed to escalating doses of *Streptococcus pneumoniae* in the nasopharynx to determine optimal dosage for establishment of nasal carriage. We will closely monitor study participants to ensure safety and tolerability of study procedures. We will measure immune protective responses to *Streptococcus pneumoniae* challenge using mucosal (nasal, throat and salivary) and blood samples to determine assay feasibility.

The study will be carried out in four stages:

1. Screening and recruitment: Potential participants will be screened to ensure health and safety as detailed in Methods.

2. Inoculation with pneumococci: Participants will be randomly allocated to inoculation with *Streptococcus pneumoniae* serotype 6B or 0.9% saline (sham inoculation) to the inside of each naris. Participants will be monitored for safety and establishment of carriage. The dose escalation schedule is described in Figure 2.

3. Detection of pneumococcal carriage: Nasal wash samples will be taken, according to a standardized protocol ^11^, at days 2, 7, and 14 days post inoculation. Classical microbiological culture will determine nasal colonisation with pneumococcal serotype 6B at each time point.

4. Immunology response measurements: exploratory blood, mucosal and nasal cell samples will be taken to confirm robust clinical and laboratory standard operating procedures and explore the immunological response to nasal challenge.

### Study endpoints

Primary endpoint: detection of the inoculated pneumococci by classical culture methods, at any time point, from nasal wash recovered from the participants at days 2, 7 and 14 following pneumococcal challenge.

Secondary endpoints: confirmation of robust clinical and laboratory methods for sample capture and processing. Acceptability of study and methods.

### Study setting

Clinical procedures will be conducted at the Queen Elizabeth Central Hospital Research Ward, Blantyre, Malawi. Laboratory procedures will be conducted at the adjacent Malawi-Liverpool Wellcome Trust Clinical Research Programme Laboratories (MLW).

### Recruitment target

A maximum of 36 healthy adult participants will be recruited to complete the study.

### Duration

Recruitment of all participants and follow up will be completed within 12 months. 

### Participants, schedule and timelines

We will inoculate healthy non-smoking adult participants with a well-characterised, fully sequenced penicillin-sensitive pneumococci and observe them for the development of pneumococcal carriage up to 14 days post inoculation. Study discharge will occur on day 20.

**Figure 1.**
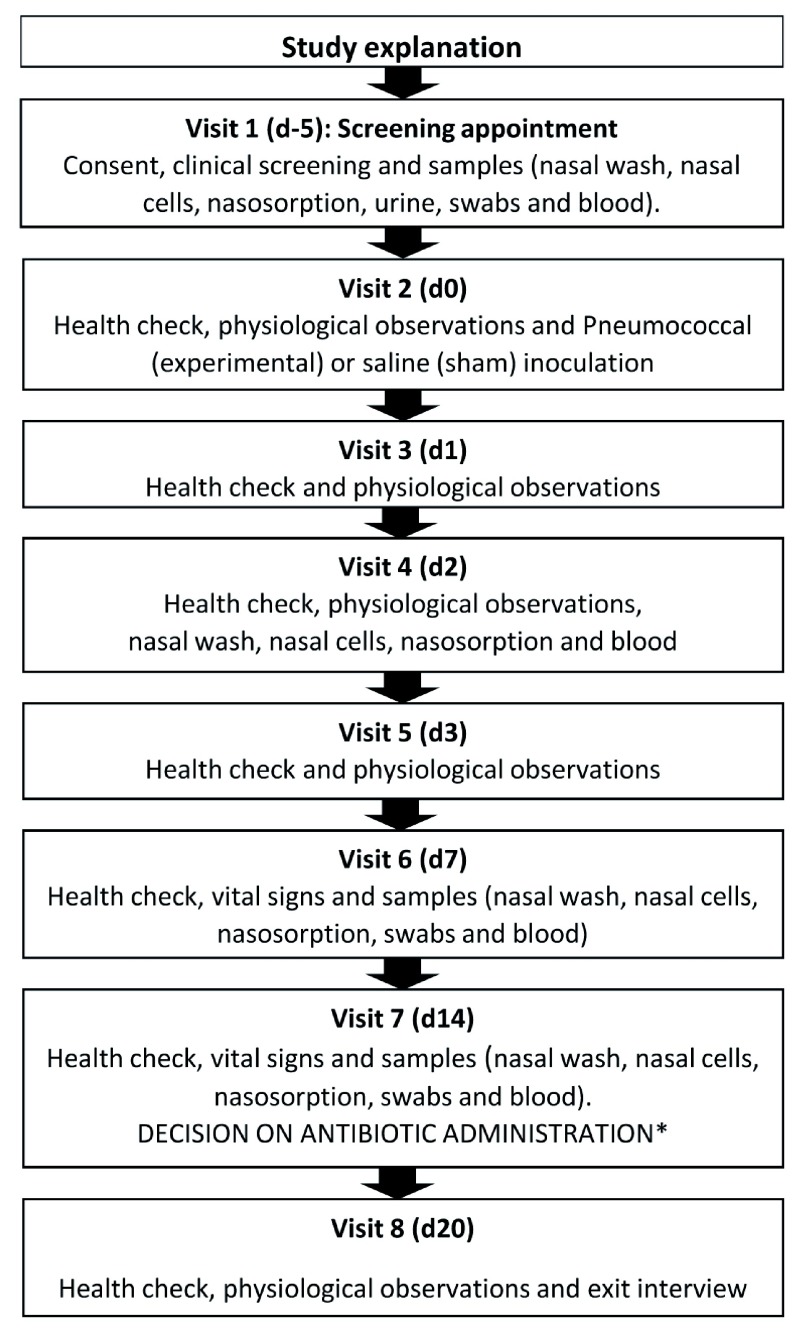
Participant flow chart. D-5 refers to 5 days before day 0, the day of inoculation, and d1 is one day after and so on. *The decision for antibiotics will be communicated directly at visit 7 except in the unlikely circumstance that only d14 is positive for carriage, in which case the result will be telephoned in time for visit 8.

Study visits must take place according to the proposed schedule to ensure participant safety. This will be clearly explained to the participants during recruitment and consent. If a participant is unable to comply, they will not be recruited (Participant flow chart - Figure 1).

## Methods

### Participant recruitment

Advertisements inviting healthy participants to participate will be widely placed. Areas will include physical notice boards, table display, electronic notice boards, the intranet/internet of local universities and colleges (with permission), social media, the local press, television and radio. Staff from the MLW team will be permitted to participate providing they are not members of the study team.

Interested persons will be asked to contact the research team by phone, email or text for further information. Once potential participants who are interested have contacted the research team, they will then be invited to meet a member of the research team. The research team will explain the study at the meeting and the Participant Information Sheet will be provided. Participants will then consider if they wish to continue (see Figure 1).

At visit 1, the informed consent process will be conducted by either the research nurse or research doctors who are formally delegated by the Chief Investigator, trained in GCP, consent and the trial protocol. The following consent, inclusion and exclusion criteria will be applied as below.

### Inclusion criteria

- Adults aged 18–40 years - ages chosen to minimise the risk of pneumococcal infection
- Fluent spoken and written Chichewa or English - to ensure a comprehensive understanding of the research project, their proposed involvement and communication with all members of the research team

### Exclusion criteria

- Previous pneumococcal vaccination
- HIV-infection: HIV testing will be performed by trained personnel certified in HIV Testing Services (HTS). “Determine” or “Unigold” will be used as the first line tests. Any participants identified as HIV-infection positive will be referred to the governmental system for infection confirmation, treatment and follow up.
- Close physical contact (e.g. sleeping in the same room or nursing) with at risk individuals (children under 5 years age, immunosuppressed adults, elderly, chronic ill health)
- Allergy to penicillin/amoxicillin
- Acute illness
  ○ Current illness
  ○ Acute illness within 3 days prior to inoculation
  ○ Antibiotic treatment within 2 weeks of inoculation

- Chronic illness that may impair immune response or impair ability to comply with study procedures and safety monitoring (e.g. HIV, diabetes).
- Taking immunosuppressive medication that may include but are not limited to steroids and steroid nasal spray.
- Pregnancy - minimise risk of pneumococcal disease
- Involved in another clinical trial unless observational or in follow-up (non-interventional) phase
- History of drug or alcohol abuse. This is very difficult to ascertain in a history therefore we will exclude people reporting drinking alcohol more than twice per week.
- History of Smoking
  ○ Current regular smoker (smokes daily/smokes > 5 cigarettes per week) - minimise risk of pneumococcal disease
  ○ Recent smoker i.e. within the last 6 months - minimise risk of pneumococcal disease
  ○ Ex-smoker with a significant smoking history (>10 pack years) – minimise risk of pneumococcal disease
- Unable to give informed consent
- In case of any uncertainty or concern, the PI will take clinical responsibility for the decision.

### Screening and preliminary assessment

Following consent, inclusion and exclusion criteria, data and samples will be collected.

• Clinical examination - the initial clinic visit will include a focused clinical history and targeted clinical examination involving auscultation of the lung fields and heart sounds. Participants will be informed if their clinical examination is normal. Should a previously unrecognised abnormality be identified, this will be explained to the individual, and appropriate investigations and follow-up will then be arranged by the study team. Further participation will be determined at the discretion of the study doctor dependent on the nature of the abnormality detected. If the participant is not eligible due to an acute illness, they may be re-screened at a later date with their verbal consent.

• *Nasal wash* - will be performed using our published method ^11^. This is a well-used and validated technique to collect nasal bacterial specimens used successfully by the Liverpool CHIM team who will rigorously train the Malawi team. Briefly, 5ml of sterile saline is instilled and held for a few seconds in the nares before being expelled into a sterile Galli pot. This is repeated twice in each naris using 20ml saline in total. In the event of nasal wash loss (defined as cough/sneeze/swallow), the procedure may then be repeated to obtain an adequate specimen. Should the nasal wash reveal natural colonisation of pneumococcus, the participant will continue in the study and receive the inoculation as per protocol, unless the natural serotype is identified as 6B, in which case the patient then leaves the study at day 2. All pneumococcal carriage negative participants will continue in the study.

• *Nasosorption strips* - Nasosorption will be obtained before a nasal wash and/or inoculation. The strips are like blotting paper developed by Hunts Development Ltd (UK, catalogue number: NSFL-FXI-11). They collect concentrated nasal lining fluid before the nasal wash to measure inflammatory responses induced by infection that may be associated with increased colonisation density and acquisition. Concentrate nasal fluid will be used to measure cytokine levels by multiplex bead array. Blotting paper will be held inside the nostril for up to 3 minutes until soaked, removed and placed in the microcentrifuge tube for storage.

• *Nasal Cells* – will be collected using a nanosampling method in which cells are obtained through minimally invasive superficial nasal scrape biopsies (rhinoprobe). Work from our Liverpool team demonstrates that participants can be biopsied multiple times with no significant side effects. Nasal cell samples will be obtained after nasal wash samples have been taken. Up to 4 samples (2 per nostril) will be obtained at each nasal sampling visit. If the researcher finds that the sample is insufficient, for example no cells are visible on the rhinoprobe, the sample can be repeated immediately. The rhinoprobes are placed in transport media immediately after collection.

• *Blood* – blood samples will be obtained using venepuncture by an appropriately trained team member. In the entire duration of the study, a maximum of 100mL of blood will be collected across the entire study for analysis including a full blood count. Participants with an abnormal full blood count will be removed from the study as determined by the clinical team (result day 2).

• *Throat swabs* – simple posterior pharyngeal swabbing on a dry swab.

• *Saliva* – salivette held for saturation or a spit in the tube.

• *Urine Sample* – for a pregnancy test as part of the safety screening. 

### Visit 2 Pneumococcal inoculation

***Preparation of bacterial stock and inoculation***

• Initial preparation and transfer to MLW - Microbiological and sequence confirmation of the purity of the inoculum will take place at LSTM and reference Public Health England laboratories prior to the onset of the study. Once complete, the microbiologically and genetically confirmed *Streptococcus pneumoniae (S. pneumoniae)* capsular serotype 6B strain BHN418 will be transferred from the LSTM to MLW. Microbiological and serotype confirmation will take place at MLW upon receipt.

• Preparation of bacteria for carriage studies at MLW – LSTM will provide MLW with mid-log broth cultures of pneumococcus for this feasibility study, these will be frozen at -80°C in aliquots of glycerol-enriched media. Immediately prior to participant inoculation, aliquots will be thawed, washed twice, and re-suspended in sterile saline at an appropriate density for each inoculation dose. The LSTM Standard Operating Procedure (SOP) will be used.

• Confirmation of accuracy - Following culture by the laboratory team at MLW, aliquots of the bacterium will be returned to the UK, as a quality control measure, to confirm the identity, purity and penicillin sensitivity of the isolate. These bacterial isolates will be sequenced in the UK to confirm identification.

• Regulatory approval - *S. pneumoniae* transfer will be reported to the Malawian Pharmacy, Medicines and Poisons Board (PMPB, national regulatory authority) prior to initiation of the feasibility study. PMPB will review the protocol as part of NHSRC, and have advised that the UK Medicines and Health care products Regulatory Authority (MHRA) position on EHPC (not an investigational medicinal product) will be recommended.

***Inoculation***

**Figure 2.**
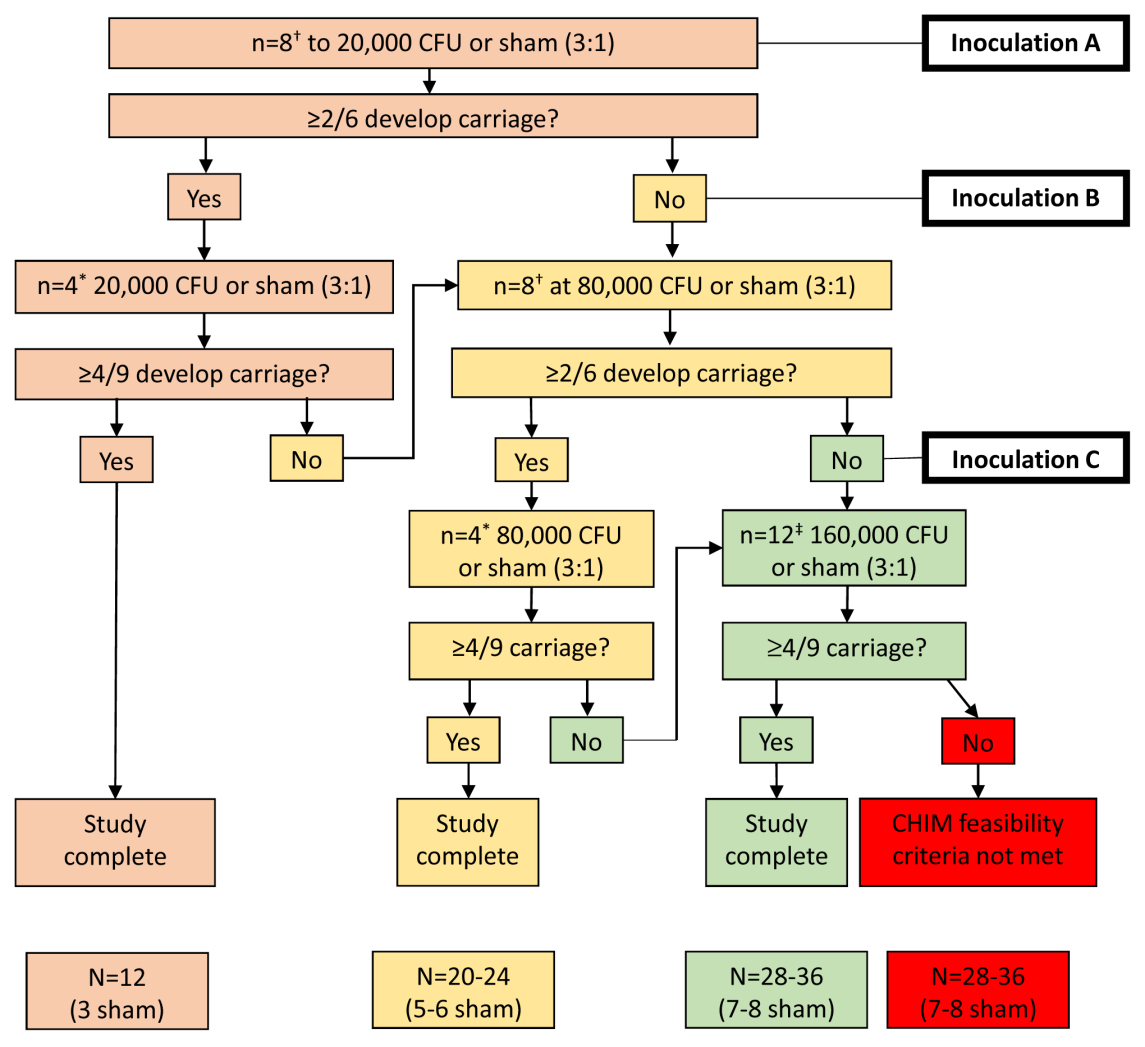
Dose escalation study design, adapted from Waddington *et al*. ^12^. The doses for Groups 1, 2 and 3 will be 20000, 80000 and 160000 pneumococcal colony forming units (CFU) per naris respectively. All participants will be monitored for adverse effects and for pneumococcal carriage. The first cohort will be inoculated with 20,000 CFU/naris ( **Inoculation A**). **†**: Eight participants will be randomised 3:1 to pneumococcal inoculation (n=6) or sham inoculation with 0.9% saline (n=2). If ≥2/6 participants from the initial cohort of eight develop experimental pneumococcal carriage, a further 4 participants will be recruited to this dose cohort. *: Four participants will be randomised 3:1 to pneumococcal inoculation (n=3) or sham inoculation with 0.9% saline (n=1). If the combined carriage rate of the completed cohorts is ≥4/9 (6+3 nasally challenged with pneumococcus), then the nasal challenge dose will be established, and we will conclude the study. If at either of these decision points, the carriage rate is insufficient, the algorithm will be restarted with a higher challenge dose of 80,000 CFU/naris ( **Inoculation B**) and the recruitment schedule repeated. If at 80,000 CFU, the carriage rate is insufficient, then an additional cohort of participants will be recruited to the 160,000CFU/naris dose escalation group ( **Inoculation C**). **‡**: 12 participants will be randomised 3:1 to pneumococcal inoculation (n=9) or sham inoculation with 0.9% saline (n=3). We have previously demonstrated 50-60% carriage rates with the 80,000 CFU 6B dose in the Liverpool pneumococcal controlled human infection model ^6, 13^.

• Inoculation - using a P200 micropipette, 0.1ml saline containing the desired dose (see Figure 2) of pneumococcus will be instilled into the nose. The participant will be seated in a semi-recumbent position. After inoculation, the participant will remain in this position for up to 15 mins. They will be given a post-inoculation advice sheet (including emergency contact details), thermometer, a course of amoxicillin (500mg TDS for 3 days) and a daily symptom log to complete.

• Dose escalation and sham inoculation - We have adopted a step-wise approach to escalating the inoculation dose. The protocol is designed to: a) minimise the possibility that we try repeatedly to attain carriage at a dose in which it is unlikely to occur; b) maximise safety by inoculating small groups (in case adverse events occur) before continuing on to larger groups in which we will be able to give reasonable precision to estimate carriage rates. Groups 1, 2 and 3 will receive increasing doses of pneumococcal inoculation as explained in Figure 2. Briefly, 20,000 cfu/naris (Group 1) is not expected to achieve carriage. If 80,000 cfu/naris achieves more than 4 from 9 participants with carriage, then the study will end at that point. Sham inoculation with 0.9% normal saline has been incorporated into the study design to assess whether any potential observed effects in participants are pneumococcal challenge-related or simply reflect the study conditions. Participants will receive only one inoculation dose. If carriage is not achieved after inoculation, participation will be concluded upon completion of visit eight.

### Randomisation

Randomization has been included to prepare (test feasibility) for future vaccine studies. The clinical team and participants will be blinded to the allocation to avoid bias in the collection and evaluation of data during the study. The randomisation code will be produced by the MLW Statistical Support Unit (co-investigator Henrion) using a computer-generated pseudo-random permutation procedure. Prior to the start of the study, a copy of the randomisation code will be supplied to the MLW laboratory team using sealed envelopes to enable the production of *Streptococcus pneumoniae* inoculations and sham inoculations as required by the randomisation schedule. The laboratory team will not be blinded to the challenge allocation, this measure is to ensure participant safety and monitoring during follow up. Research team members will be assigned solely to one of two separate delegation logs for blinded and unblinded activities (clinical and laboratory).

### Determination of colonisation

Colonisation will be defined by the microbiology result of nasal washes taken at 2-, 7- and 14-days post inoculation. Nasal washes will be plated on to culture media and incubated overnight at 37°C in 5% carbon dioxide (CO _2_). Colonies will be confirmed as *S. pneumoniae* using classical microbiological techniques including (i) typical draughtsman-like colony morphology, (ii) the presence of α-haemolysis, (iii) optochin sensitivity and (iv) Gram-positive diplococci. Typing by latex agglutination will be done using a commercial kit to confirm pneumococcal serogroup. Isolates will be frozen at -80 ^o^C for storage. Results from the cultured nasal wash will also be confirmed using polymerase chain reaction (PCR) based methods of bacterial detection.

Monitoring of colonisation – monitoring of colonisation will be performed by microbiology analysis of nasal washes.

### Safety during colonisation

A three-day course of amoxicillin and a digital thermometer will be given on the day of inoculation. These will be carried by the participants for immediate treatment after phone consultation should the participant develop any symptoms as described on the participant safety advice leaflet ^14^. Participants will be required to make text message/phone contact with a specified member of the research team before 1200 hrs every day for 7 days post-inoculation *regardless of whether they have symptoms or not*. Should they not make contact by the specified time, a member of the research team will contact the participant. If no contact is made, then a prior defined ‘secondary contact’ will be telephoned. During the post-inoculation period, participants will have access to a 24/7 on-call telephone service until the end of the study.

### Termination of carriage

All study participants who are nasally colonised with pneumococcus at the end of the study will be asked to take oral amoxicillin 500mg three times daily for 3 days. This will be communicated at visit 7, or by telephone in the unlikely event of only visit 7 samples (day 14) showing carriage.

### Immunological assays

• *Antibody titres* – measurement of pneumococcal-specific protein and polysaccharide antibodies will be conducted on nasal wash, salivary and serum samples using ELISA as previously reported ^15^. This will allow us to test the existing LSTM protocols for the identification of potential antibody correlates of protection against pneumococcal colonisation.

• *Cellular responses* – measurement of pneumococcal-specific memory B and T cells will be performed on peripheral blood and nasal samples using flow cytometry and ELISPOT as previously reported ^16, 17^. This will allow us to test the existing LSTM protocols to identify potential cellular correlates of protection against pneumococcal colonisation.

• *Cytokine profiles* – measurement of cytokines will be done on nasosorption and serum samples using multiplex bead array as previously reported ^18^. This will enable us to identify potential soluble marker correlates of protection against pneumococcal colonisation.

• *Host transcriptomic analysis* – measurement of host nasal mucosa transcriptomic profile will be done using RNA sequencing (RNAseq) on nasal cells and peripheral blood as previously reported ^16^. This will allow us to establish protocols for characterising potential nasal or blood transcriptomic signatures associated with protection or susceptibility to pneumococcal carriage.

• *Microbiome profiles* – measurement of microbiome profiles will be done on throat swabs and nasal washes using next-generation sequencing and metagenomic analysis as previously reported ^18^.

### Sample size justification

This is a feasibility study that transfers a proven existing technique therefore we have not formally calculated power to detect the carriage rate. The study protocol and sample size are based on our previously published study demonstrating 50% nasal carriage at the 80,000 CFU dose in Liverpool, UK ^13^. This feasibility study is designed to determine if experimental pneumococcal nasal carriage is possible in the Malawian setting.

The primary endpoint is the occurrence of pneumococcal colonisation determined by the presence of pneumococcus in nasal wash samples at any time point post inoculation detected using classical microbiology.

### Statistical analysis

The reporting of this study will be prepared in accordance to the CONSORT 2010 ^19^ guidelines. The full statistical analysis plan is provided as extended data ^20^. A CONSORT diagram will summarise participant screening, enrolment, randomisation, inoculation, withdrawals, follow-ups and analysis. All continuous data variables will be summarized using the following descriptive statistics: N (size of relevant analysis population), n (size of analysis population without missing values), mean, standard deviation, median, 25 ^th^ percentile value, 75 ^th^ percentile value and interquartile range, minimum and maximum. The proportion of observed levels will be reported for all binary and categorical measures. When appropriate, corresponding exact, binomial 95% confidence intervals (CIs) for proportions will be included.

***Timing of analyses.*** The primary feasibility endpoint of nasal colonisation will be assessed at least 2 times and up to 5 times given the adopted dose escalation design ( Figure 2), after all participants from each inoculation batch have concluded visit 7 (d14). All other feasibility analyses will be conducted at the completion of the study. Safety endpoints will be monitored continuously throughout the study and will be summarised at the completion of the study.

***Missing data.*** Due to the close monitoring of study participants, with secondary contact details available for every participant, few missing data are anticipated. Still, study participants may withdraw from the study, and missing data may originate due to a variety of reasons.

For the primary feasibility endpoint, data will be available for as many participants as given by the study design from Figure 2 since an alternative participant will be recruited in the case of withdrawal of a participant. Participants missing study visits so that no day 2, 7 or 14 data is available will be withdrawn from the study and their data excluded from data analysis. A new participant will be recruited in such a case and this new participant will be allocated to the same pneumococcus / sham inoculation as the participant ID for the withdrawn participant was randomised to. The primary feasibility endpoint will be assessed, at each of the five analysis time points using colonisation results data from all participants that are required for that stage’s analysis and that have completed visit 7 (d14). Other analyses are primarily descriptive or make use of censored data techniques, so that no advanced missing data methods will be used in these analyses. Data collection forms are provided in extended data ^21^. 

***Statistical significance and multiple testing.*** Success (feasibility) of the study will be decided according to the decision rules stated on Figure 2.

Success (safety) of the study will decided by decision of the DSMB & TC upon revision of each SAE and SUSAR. For this reason, no formal null hypothesis significance testing will be performed for the main analysis and no multiple testing adjustment will need to be made.

For secondary analyses, statistical significance is defined as p<0.05, but effect sizes / parameter estimates, exact p-values and confidence intervals will be stated. While we will report on statistical significance, results will be presented and discussed in accordance with recent guidance ^22^.

***Technical details.*** All analyses and figures will be performed using the R environment (v.3.5.3 or later) for statistical computing and graphics and associated packages ^23^.

***Feasibility.*** The primary analysis aims to establish feasibility of the Malawi CHIM: does one of the three doses (20,00, 80,000, 160,000 CFU) lead to ≥50% nasal colonisation?

To assess this, the decision algorithm specified in the study design ( Figure 2) is followed and no statistical analysis is required. However, we will complement this with a number of descriptive and secondary analyses: For each inoculation dose used in the study, we will estimate colonisation (by serotype 6B) rates and compute 95% exact, binomial confidence intervals. We will also compute this for participants inoculated with the sham, saline solution. If more than 1 dose is used in the study, we will plot the estimated colonisation rate against inoculation dose for a crude estimate of the dose-response curve. For each inoculation dose, we will also plot time curves of colonisation rates at visits 0 (d-5), 4 (d2), 6 (d7), 7 (d14). We will also use lytA qPCR to assess nasal carriage by *Streptococcus penumoniae*. We will repeat all analyses using this method of carriage detection. Further, for the qPCR results, we will estimate average carriage densities and associated 95% confidence intervals for each challenge dose. If more than one dose is used, we will also plot individual carriage densities against inoculation dose for a crude estimate of the carriage density dose response curve. We will estimate the lower limits of detection and quantification for the qPCR data, then use censored data techniques to account for the uncertainty associated with values below these limits.

We will compile a summary table for demographic (age, sex), baseline physiological (pulse rate, oxygen saturation on room air, systolic blood pressure, diastolic blood pressure, tympanic body temperature, height, weights, BMI, baseline carriage of *S. pneumoniae* other than serotype 6B) and baseline immunological variables. Baseline physiological and immunological variables are those measured at the inoculation visit or for which the sample was taken at the inoculation visit (prior to the inoculation). Analogous tables will be compiled for the assessments made at visits 2–8.

***Safety.*** AEs, SAEs, SUSARs are monitored throughout the study and any decision to stop the trial is taken by the TSC in consultation with the DSMB. At study end, AEs, SAEs and SUSARs will be listed by type and by inoculation group (sham, *Streptococcus pneumoniae* 20,000 CFU, 80,000 CFU, 160,000 CFU) and we will also stratify these lists by sex and age band. Symptoms reported by study participants will also be listed, including sore throat, coryzal symptoms, earache, feverish, cough, headache, new rash and/or other. The reasons why any participants are withdrawn from the study and/or excluded from analysis will also be listed.

### Safety considerations and assessment

***Study design to ensure safety.*** Safety is paramount in human infection studies. While the risk to individuals of developing any infection is very low (40% Malawian adults experience natural colonisation at any time, and the incidence of invasive disease is 20/100 000 patient years ^5^), the study is designed to ensure any risk is minimal by appropriate:

- Study team selection and rigorous training in human challenge procedures
- Study design with staggered recruitment approach (sample size and dose)
- Careful serotype selection and dosing based on 10 years of LSTM experience
- Participant selection and exclusion criteria as detailed
- Participant education through a participant information sheet (provided)
- Rigorous safety procedures including daily monitoring and providing participants with a thermometer and a course of amoxicillin tablets in case of emergency ^14^
- 24-hour emergency telephone contact with researchers, including close individual daily monitoring, and access to hospital facilities and prompt treatment if required
- To further mitigate risk, participants will be provided with accommodation at the Grace Bandawe Centre for three nights immediately following nasal challenge. If a participant determines that it is not possible to stay at the Grace Bandawe Centre for three nights then a pragmatic approach to further study procedures will be taken. If the participant can demonstrate that they remain readily available by phone and accessible with transport, this need not affect the procedure. Twice daily checks could replace the resident period. In the Liverpool CHIM there is no resident period at all. If, however, the participant absconds or is intending to be uncontactable, then participation in the study will be stopped and antibiotics offered as at the end of the protocol.

We will schedule inoculations such that participant monitoring (to day 20) is completed at each pneumococcal dose (20,000 and 80,000) prior to escalation. The feasibility study research investigators Gordon, Jambo, Morton and Rylance have successfully worked together to safely deliver pneumococcal CHIM studies in Liverpool and will transfer this knowledge and experience to MLW. No episodes of pneumococcal infection or serious unexpected serious adverse reactions (SUSARS) have occurred in any of our participants whether they had no carriage, natural carriage or experimental carriage. We will transfer and rigorously implement the Standardised Operating Procedures to ensure safety for Malawian participants.

***Participant safety procedures.*** We will provide all participants with a safety information sheet ^14^. This sheet classifies symptoms into mild, moderate and severe. We will take a proactive approach for participants, with daily contact throughout the study and enquiry about development of any symptoms. We will also encourage participants to report development of any symptoms with 24-hour medical cover throughout the study visit schedule. In the event of illness, we will apply standardised operating safety procedures for participants. The research team will pay for any costs associated with these procedures and participants will incur no out of pocket expenses.

*Mild illness*

Mild illness is defined as development of any new symptom that is of concern to the participant during the study period. These may include mild coryzal symptoms such as blocked or runny nose. Participants will be encouraged to contact the research team by telephone within office hours. During the telephone assessment participants will be screened for potentially concerning moderate and severe symptoms. If there is any concern, participants will be invited to the research clinic for a detailed clinical assessment by one of the study doctors. The assessment will be fully documented within study records including rationale for any treatments and/or required modifications to study procedures.

*Moderate Illness*

Moderate illness events are defined as fever with temperature >37.5°C, shivering, headache, new rash, drowsiness, cough, new earache and or new eye infection. Participants will be clearly instructed to call the research team at any time of the day upon development of these symptoms. During the telephone assessment participants will be screened for severe symptoms and requested to attend either the research clinic (office hours) or Mwaiwathu hospital (outside of office hours) for medical assessment. If severe symptoms are identified on telephone assessment, participants will be asked to take antibiotics from their supplied emergency pack and attend Mwaiwathu hospital. Study doctors will assess the participants at either the research clinic or Mwaiwathu and offer appropriate treatments. The assessment will be fully documented within study records including rationale for any treatments.

*Severe illness*

Severe illness events are defined as any symptoms that cause serious concern to participants following inoculation. We have purposefully taken a conservative approach to encourage participants to seek early medical advice and treatment if they are concerned. Participants will be counselled as to what symptoms the study team may be particularly concerned about during the consent process ^24^. These would include any potential symptoms of sepsis. In the event of severe illness, participants are requested to start taking the provided emergency antibiotics, directly attend Mwaiwathu hospital and contact the study team. A study doctor will attend the patient in Mwaiwathu and offer appropriate treatments. The assessment will be fully documented within study records including rationale for any treatments.

### Evaluation of adverse events (AE) and serious adverse events (SAE)

***Adverse events.*** Non-serious adverse events will be collected systematically during the research and recorded in the case report form. Participants will keep a log of symptoms and this will be summarised and reported to the DSMB.

*Serious adverse events*

Any SAE as defined in ICH-GCP occurring to a research participant will be reported to the DSMB, NHSRC and study sponsor within 24 hours of the study team becoming aware of the event. In this event, the research will be stopped temporarily for investigation and any further work deferred until DSMB and NHSRC advice has been provided to the TSC for consideration.

***Risks to researchers.*** These are standard clinical (needle stick) and laboratory (biohazard) risks. Experienced staff will carry out procedures that are within their competencies in accordance with standard operating procedures regulated by good clinical practice and national guidelines. Appropriate risk assessments are in place for all laboratory SOP. All laboratory work will be conducted in an appropriately rated laboratory in line with health and safety regulations for research with human tissues / infectious agents.

***The Data Safety Monitoring Board (DSMB).*** The DSMB will be established prior to commencing this study and include members who are experts in human infection studies, safety and statistical procedures. The DSMB will monitor the study and advise the TSC, including the PI and study team. Briefly, all SAEs will be reported to the DSMB and study sponsor within 24 hours and recruitment/inoculation paused pending DSMB review and recommendation to the Trial Steering Committee (TSC) in line with GCP guidelines. The study team will provide at least monthly updates (by email) on all recruitment to the DSMB. The DSMB will meet formally (by telephone conference) biannually and in the event of any SUSARs.

***Stopping criteria.*** This study will pause in the event of a serious adverse event or on completion of the protocol. Thereafter, DSMB and NHSRC members will assess the event before advising the Trial Steering Committee on measures required to resume the study or if the study should be stopped. The study will only be resumed if both the DSMB and NHSRC are satisfied that required measures are in place. If the DSMB or NHSRC recommends the early stopping of the study, all reasons must be stated clearly. If there are DSMB or NHSRC members with dissenting views, these must be appended to the recommendations. If there are no trial related serious adverse events, the study will stop upon completion of the protocol (see Figure 2).

### Ethical considerations

***Autonomy.*** The participants will be given high-quality information that is written and spoken using common lay terms without jargon to allow them to understand the research objectives and the risks and benefits of all procedures ^24^. They will then be given time to consider the information before consenting to any involvement. At no stage should the participant feel pressured or persuaded into participating in the research study.

Participants have the right to withdraw their consent and therefore withdraw from the study at any time without giving reason. If a participant withdraws from the study, we will recruit an alternative participant to complete the study as per Figure 1.

***Non-maleficence.*** Inoculation of *S. pneumoniae* will be as per the established and safe LSTM protocols and will be performed by highly trained staff with close supervision (24hr on call access to medical professionals involved in the study) and follow-up. Specific inclusion and exclusion criteria are set to further protect the participant. Experience and trained research staff will perform venepuncture and nasal sampling.

***Beneficence.*** Participants will receive a health check including HIV-infection status assessment when taking part in this study. In addition, participants may benefit from a better understanding of clinical research, they may also benefit from a sense of contributing to medical research in a valuable way. Participants will be remunerated for their time and inconvenience – discussed below.

***Justice.*** This must be balanced with non-maleficence. Inclusion and exclusion criteria are in place primarily to protect individuals from undue risk.

### Ethical approvals

Ethical approvals for this study have been granted in country by the NHSRC (Approval number: 19/08/2246) and from an LSTM institutional perspective (Approval number: 19-017).

### Study sponsorship

LSTM will sponsor the study (Approval number: 19-017). The Malawian National Commission for Science and Technology has confirmed regulatory endorsement of no-fault insurance cover for participants in this study and granted permission for the implementation of the study (Reference: NCST/RTT/2/6).

### Remuneration

It is intended that financial factors will not significantly influence an individual's decision to participate in this study. We reimburse participants for out of pocket expenses such as travel, and compensate time spent and burden. The sums offered in this study are consistent with remuneration guidelines published in Malawi, paid pro-rata (per activity and not dependent on study completion) ^25^.

If a participant withdraws from the study early, they will be compensated for the parts they took part in up until the time they withdrew. Payments are summarised in Table 2: MK 8400 will be paid at the end of each study visit (at time of writing this amount is equivalent to £8.90 or $10.50 per visit for eight visits).

**Table 2.** Research Participant Remuneration. *Accommodation and board costs will be paid directly by the research team to the Grace Bandawe Centre. Visits 3, 5 and 8 mild discomfort. Visits 1, 2, 4, 6 and 7 mild/moderate discomfort. Exchange rate is approximately 800 Malawian Kwacha to one US dollar.

MARVELS Research Participant Remuneration (based on Malawian guidelines ^25^			
Reimburse expenses	Rate in MK	Number of events	Total
a) Transport	900	8	7200
b) Subsistence (one meal)	1500	8	12000
c) Accommodation (one night) *	15000	0	0
Compensation			
Total time travelling (hrs)		8	
Total time in research facility (hrs)		24 research 72 accommodation	
Time in days (day = 8 hours)	1000	12	12000
Procedure A (mild discomfort)	2000	3	6000
Procedure B (moderate discomfort)	6000	5	30000
Procedure C (long or complex)	10000	0	
TOTAL for study			67,200
AVERAGE per visit			8,400

### Confidentiality and anonymity

Only authorised members of the research team will have access to any personal information. Only information of direct relevance to the study will be collected. All electronic records containing personal information will be stored in a password protected database on a password protected server at MLW. Electronic case report form data will be collected on encrypted study-specific tablet devices and synchronised daily onto the MLW server. This approach is well established at MLW, and the policy governing MLW data management policy is available upon request. Paper documentation containing personal information will be kept in a locked filing cabinet in a locked room in the Queen Elizabeth Central Hospital research clinic.

Each participant will be assigned a unique non-identifiable study number by a member of the clinical research team at recruitment. Unlinked non-identifiable clinical data will be stored and analysed at the MLW-laboratories and collaborating laboratories.

### Samples and data

***Sample and data storage.*** MLW will act as custodian for all data and samples collected during the study. Consent will be obtained from the participant to use the samples for this research only. Samples will be stored for a maximum of five years. 

***Collaborating laboratories.*** Samples will also be sent to national and international collaborating laboratories (MTA attached with application) to utilise specialist expertise not in Malawi. Samples will be labelled with the anonymised study number. Consent will specifically be obtained from the participants to allow samples to be sent to our collaborators ^24^.

### Assessing acceptability

We need to understand acceptability of study procedures to participants to inform future CHIM study design. Feedback from participants will help to identify any areas where study design could be adjusted to ensure participants have a positive experience and to maximise their comfort. Brief exit interviews will be carried out with each participant at visit 8. Data collection will examine reasons behind decisions on participation; understanding of the trial purpose, procedures and risks and views on information provision; satisfaction with trial experiences; and any social impacts of participation (e.g. impacts on education, household relationships, livelihoods). Emerging findings will be communicated to the clinical team to inform ongoing procedures, with quick communication of areas needing immediate attention.

### Dissemination of findings

The findings from this study will be disseminated amongst the scientific community. We intend to publish our findings in peer reviewed scientific journals and present data at appropriate local, national and international conferences. Authorship for any submitted manuscripts will follow ICMJE guidelines. We will produce a close-out report for the NHSRC at the end of the study and a final report once data are published. In addition, we will produce a lay report of our findings which will be made available to all participants.

### Study status

At time of manuscript submission (23 ^rd^ January 2020), the study has commenced recruitment and has currently inoculated eight participants at the 20,000 CFU dose

### Sponsorship and indemnity

Sponsorship - The Liverpool School of Tropical Medicine (LSTM) will act as sponsor

Indemnity **-** Clinical Trials Insurance has been obtained for this study through LSTM

### Amendments

Future amendments to the protocol will require approval by the National Health Research Ethics Committee and the Sponsors.

### Future development of the MARVELS programme

Future planned studies include challenge of participants with pneumococci after vaccination with PCV13 (pneumococcal conjugate vaccine) to determine how this vaccine impacts on nasal carriage in a Malawian population (pending future application to and approval by ethics committees). This study will be based on our previously completed and published randomised controlled trial in a UK population ^6^. The overarching aim of these studies is to build the best possible model to support scientific investigation and vaccine development in Malawi.

## Discussion

Pneumococcal disease is a persistent public health problem, particularly in low income countries ^26^. The introduction of PCV13 has reduced the risk of invasive disease for vaccinated individuals but does not prevent nasal colonisation with “vaccine type” pneumococcal serotypes in the African setting ^8^. Persistent nasal carriage despite vaccination ameliorates the anticipated herd immunity impact of PCV13, leaving vulnerable non-vaccinated individuals at risk of pneumococcal disease. There is therefore an urgent need to accelerate development of more effective vaccines that prevent nasal colonisation. To date, pneumococcal vaccine candidates have been tested in high income settings on individuals at low risk of disease. We will transfer safe and standardised procedures from the experimental human pneumococcal carriage group in Liverpool, UK to establish this controlled human infection model in Blantyre, Malawi. Our feasibility study will determine if these procedures can be safely and effectively implemented within the Malawian context. Nested within the study, the social science team will explore participant and community acceptability to inform how best to conduct these studies in the future. Once established, we will be in a position to test and select vaccine candidates tailored to an at-risk population. Concurrently, we will investigate underpinning immunological mechanisms for disease susceptibility to inform the discovery and development of new vaccine candidates.

## Data availability

### Underlying data

No data are associated with this article.

### Extended data

Harvard Dataverse: EHPC Safety Leaflet. https://doi.org/10.7910/DVN/3LEAYH ^14^

Harvard Dataverse: MARVELS 1.0 Statistical Analysis Plan. https://doi.org/10.7910/DVN/SGJ2IU ^20^

Harvard Dataverse: MARVELS Feasibility Data Collection Forms. https://doi.org/10.7910/DVN/Y2AWT8 ^21^

Harvard Dataverse: MARVELS feasibility Participant information and consent forms. https://doi.org/10.7910/DVN/WNBR96 ^24^

### Reporting guidelines

Our feasibility study is not registered as a randomised controlled trial however, where applicable, we have used the SPIRIT guideline checklist to address recommended items.

SPIRIT checklist for ‘A pneumococcal controlled human infection model in Malawi: Transfer of an established pneumococcal carriage model from Liverpool, UK to Blantyre, Malawi – A feasibility study’ https://doi.org/10.7910/DVN/NLS7HB ^27^

Data are available under the terms of the Creative Commons Zero "No rights reserved" data waiver (CC0 1.0 Public domain dedication).
