# Supplementary material for: A pneumococcal controlled human infection model in Malawi: Transfer of an established pneumococcal carriage model from Liverpool, UK to Blantyre, Malawi – A feasibility study
Source: Wellcome Open Res. 2020 Apr 14;5:25. Originally published 2020 Feb 11. [Version 2] doi: 10.12688/wellcomeopenres.15689.2 (PMC7194502; doi:10.12688/wellcomeopenres.15689.2)
Supplement: Peer review history

# Peer review: Reviewer response for version 2

**DOI:** 10.21956/wellcomeopenres.17378.r38430

**Published:** 2020-04-28

**Authors:** Rodrigo Oliveira

**Affiliations:** 1 Oswaldo Cruz Foundation, Rio de Janeiro, Brazil

The authors have clarified my comments addressing all the points raised.

Is the study design appropriate for the research question?

Partly

Is the rationale for, and objectives of, the study clearly described?

Yes

Are sufficient details of the methods provided to allow replication by others?

Partly

Are the datasets clearly presented in a useable and accessible format?

Not applicable

Reviewer Expertise:

Immunology, molecular immunology, immunoepidemiology

I confirm that I have read this submission and believe that I have an appropriate level of expertise to confirm that it is of an acceptable scientific standard.

# Peer review: Reviewer response for version 1

**DOI:** 10.21956/wellcomeopenres.17194.r37870

**Published:** 2020-03-25

**Authors:** James E. Meiring

**Affiliations:** 1 Oxford Vaccine Group, Department of Paediatrics, University of Oxford, Oxford, UK

This is a detailed and well written protocol for the set-up of a pneumococcal controlled human infection model, taking existing protocols from a UK university and transfering them to Blantyre, Malawi.

 The rationale is well explained, outlining the difference in carriage rates between high- and low-income countries, and the common problem of vaccine development and testing being distant from the settings where they are required to have the greatest impact.

 The explanation for dose-escalation is clear, and the clarification to reviewer one is helpful. The detail for further laboratory analyses using these samples also justifies the development of CHIM in Malawi.

Is the study design appropriate for the research question?

Yes

Is the rationale for, and objectives of, the study clearly described?

Yes

Are sufficient details of the methods provided to allow replication by others?

Yes

Are the datasets clearly presented in a useable and accessible format?

Yes

Reviewer Expertise:

Epidemiology, Salmonella Typhi, Clinical Vaccine Trials, Controlled human infection models

I confirm that I have read this submission and believe that I have an appropriate level of expertise to confirm that it is of an acceptable scientific standard.

# Response

**Published:** 2020-04-03

**Authors:** Ben Morton

**Affiliations:** Liverpool School of Tropical Medicine, Malawi

Thank you for your review. In particular for addressing the clarification we offered to reviewer one on the issue raised.

# Peer review: Reviewer response for version 1

**DOI:** 10.21956/wellcomeopenres.17194.r37872

**Published:** 2020-03-04

**Authors:** Rodrigo Oliveira

**Affiliations:** 1 Oswaldo Cruz Foundation, Rio de Janeiro, Brazil

The paper presented is related to the protocol to be used in a controlled human infection study to be developed in Malawi. The background is well described as well as the rationale for the development of the study. The paper describes how the investigators propose to transfer the technology developed at the Liverpool School of Tropical Medicine (LSTM) to the Malawian laboratory for the performance of the study.

 The aims of the feasibility study proposed are: 

 1) Establish *Streptococcus* *pneumoniae* experimental human pneumococcal carriage in Malawi; 2) Confirm optimal nasopharyngeal pneumococcal challenge dose (this is important since colonization of the nasopharynx is a prerequisite for pneumococcal disease); 3) Confirm safety and measure potential symptoms; 4) Confirm sampling protocols and laboratory assays; 5) Assess feasibility and acceptability of consent and study procedures. It is expected that at the end of the feasibility study the researchers will be able to use CHIM to target new pneumococcal vaccine candidates in a setting of populations at risk. The model developed at LSTM has been successfully used in as normal non-exposed volunteers. It is well argued by the authors that the transfer and implementation of CHIM will accelerate the identification of vaccine(s) that are suitable for a population at risk. The arguments are well supported by the authors and clear outcomes described.

 In the Malawian study, the researchers plan to use three different doses of pneumococcal inoculation (20,000; 80,000 and 160,000) as described in the protocol. Most of the protocol is well presented and clear to understand. The main concern is related to the figure 2 that describes the protocol of the dose escalation study design that was adapted from a previous study by Waddington et al (2013) using *Salmonela typhi*. The concern has to do with the inoculations. The first group to will receive the 20,000 CFU dose and evaluate for development of carriage. If the volunteer does not develop carriage he/she is moved to the group that will receive 80,000 CFU. The same inclusion of non-carriages will be moved to the 160,000 CFU. It seems to me that this will lead to an increase in dose of on a challenge dose in individuals that are moved from one group to the other. Even if carriage does not occur, it is quite possible the immune response be activated since mucosal immune response is significantly faster, mainly the innate immune response. It is not clear whether the authors have evaluated this possibility at LSTM in non-exposed volunteers. It is important to make it clear since the study will be carried in exposed populations. A clear description of these possible limitations would be highly relevant for the clear understanding of the protocol.

Is the study design appropriate for the research question?

Partly

Is the rationale for, and objectives of, the study clearly described?

Yes

Are sufficient details of the methods provided to allow replication by others?

Partly

Are the datasets clearly presented in a useable and accessible format?

Not applicable

Reviewer Expertise:

Immunology, molecular immunology, immunoepidemiology

I confirm that I have read this submission and believe that I have an appropriate level of expertise to confirm that it is of an acceptable scientific standard, however I have significant reservations, as outlined above.

## References

[1] : An outpatient, ambulant-design, controlled human infection model using escalating doses of Salmonella Typhi challenge delivered in sodium bicarbonate solution. *Clin Infect Dis*.2014;58(9) : 10.1093/cid/ciu078 1230-40 10.1093/cid/ciu078 24519873PMC3982839

# Response

**Published:** 2020-03-07

**Authors:** Ben Morton

**Affiliations:** Liverpool School of Tropical Medicine, Malawi

Thank you for this thorough review.

In response to the reviewers’ concern about multiple doses for participants we would like to clarify that no participant will move between dosing groups. To elaborate, if a participant is inoculated at the 20,000 dose but does not develop carriage then they will complete the visit schedule as per table 1. Once completed this participant is then discharged from the study. No further doses of pneumococcus will be administered to that participant.

A maximum of 12 participants will be recruited to the 20,000 CFU dosing group. If less than four participants develop colonisation then we will move to the 80,000 CFU dosing group, recruiting up to 12 different participants. If four or more participants develop colonisation then the study will complete. This pattern is then repeated for the 80,000 CFU dose.

I hope this clarifies the reviewer’s concern but we would be happy to provide further details if anything is unclear
